# Drug toxicity assessment: cell proliferation versus cell death

**DOI:** 10.1038/s41420-022-01207-x

**Published:** 2022-10-14

**Authors:** Elena V. Sazonova, Mikhail S. Chesnokov, Boris Zhivotovsky, Gelina S. Kopeina

**Affiliations:** 1 Faculty of Medicine, MV Lomonosov Moscow State University, Moscow, Russia; 2 Division of Toxicology, Institute of Environmental Medicine, Karolinska Institute, Stockholm, Sweden

**Keywords:** Cell death, Cell growth

## Abstract

Analysis of the toxicity of chemotherapeutic drugs is one of the main tasks of clinical pharmacology. Decreased viability of tumor cells may reflect two important physiological processes, namely the arrest of proliferation associated with disturbances in cellular metabolism or actual cell death. Elucidation of the exact processes mediating a reduction in the number of cells is fundamentally important to establish the mechanisms of drug action. Only the use of a combination of cell biological and biochemical approaches makes it possible to understand these mechanisms. Here, using various lines of tumor cells and a set of methodological approaches, we carried out a detailed comparative analysis and demonstrated the possible ways to overcome the uncertainties in establishing the mechanisms of cell response to the action of chemotherapeutic drugs and their toxicity.

## Introduction

Drug toxicity is one of the key areas of interest in pharmacology that is responsible for the attrition of approximately one third of drug candidates and is a major contributor to the high cost of drug development [1]. It deals with a concept of “cell viability” that is commonly comprehended as an aggregate characteristic describing the number and proportion of living and dead cells in the population. The development of reliable, accessible, and scalable viability assays based on cell death–associated effects of biomarkers is extremely important for efficient screening of drug toxicity.

The 3-(4,5-dimethylthiazol-2-yl)-2,5-diphenyl-2H-tetrazolium bromide (MTT) assay is currently one of the most extensively used methods of drug toxicity assessment [2–4]. According to the PubMed database of medical and biological publications, about 1000 articles mentioning the method are published every month. This method, developed by Mosmann et al. [5] in 1983, is based on the fact that the activity of NAD(P)H-dependent cellular oxidoreductases can reflect the number of viable cells. These enzymes reduce the MTT dye into an insoluble formazan, which is purple and can be subsequently dissolved in dimethyl sulfoxide for colorimetric measurement [3, 5]. MTT is a tetrazolium salt consisting of a positively charged quaternary tetrazole ring core containing four nitrogen atoms surrounded by three aromatic rings including two phenyl parts and one thiazolyl ring [6, 7]. Assays that employ other closely related tetrazolium dyes such as XTT, MTS, and WST, in combination with the intermediate electron acceptor 1-methoxy-phenazine-methosulfate (PMS), are easier to use because the resulting formazan dye is soluble in water, thus allowing researchers to avoid the last dissolution step [8–11]. This assay has extensive utility for the evaluation of cell metabolic activity by which the viability of the cell can be inferred.

The reduction in the number of viable cells may result from two major processes—inhibition of cell metabolism and/or proliferation (cytostatic effect) or actual cell death (cytotoxic effect)—which should be discerned from each other. It is necessary to use a combination of methods to understand exactly what is happening in the cell. MTT and other enzymatic assays evaluate the activity of cell metabolism by estimating mitochondrial NAD(P)H oxidoreductases or cytoplasmic esterase activities [12]. Various viability/cytotoxicity assays have been developed to combine the advantages of enzyme-based assessment of the living cell number and simultaneous estimation of the number of dead cells stained with DNA-binding fluorescent dyes [13]. Flow cytometry-based Annexin V assay detects apoptotic cells that express phosphatidylserine on their surface and provides information about the percentage of viable and dead cells in the population [14, 15]. Western blotting allows estimating changes in proteins that participate in the regulation of signaling pathways associated with different cell death modalities [16]. Selective inhibitors can be used to assess the contribution of a particular form of cell death, such as apoptosis, necroptosis, or autophagy [17].

In the present study, we have compared several methodological approaches to cell viability and cell death assessment using human cancer cell lines as a model system. Based on previously obtained data, we have chosen two cell lines, SKOV3 and SW620, that display the most illustrative changes upon treatment with cytotoxic and cytostatic drugs. SKOV3 is an ovarian p53-null cell line that does not express *TP53* [18]. The colorectal adenocarcinoma SW620 cell line has a R273H mutation in the p53 protein that has a dominant-negative effect on DNA binding and p53-dependent gene expression [19]. By comparing the results obtained using different cell death evaluation techniques, we have demonstrated their possible advantages and drawbacks that should be taken into account while planning drug toxicity assessment.

## Results

### Different response of SKOV3 and SW620 cells to treatment with cisplatin and topotecan

Parameters such as the IC_50_, the half maximal effective concentration (EC_50_), or the GI_50_ are often used to measure drug sensitivity [20–22]. We treated our model cell lines with two drugs with different mechanisms of action: cisplatin (causes DNA crosslinks) and topotecan (inhibits topoisomerase I). We used the MTS assay to determine the compound concentrations required to reach 50% of maximum possible response to treatment (IC_50_) or 50% reduction in number of viable cells (GI_50_).

**Fig. 1:**
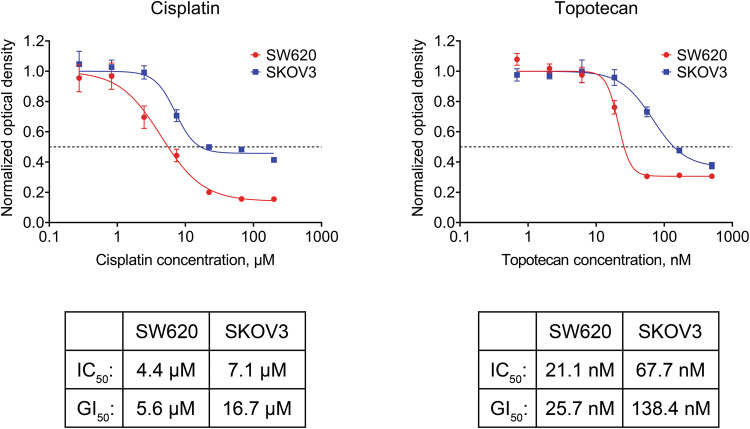
Response of SW620 and SKOV3 cells to treatment with various concentrations of cisplatin or topotecan. The MTS assay values obtained from treated cells were normalized to the untreated sample. The data are presented as mean ± standard deviation (*N* = 4 for cisplatin, *N* = 3 for topotecan). The dashed lines indicate a 50% reduction in the MTS signal.

The calculated cisplatin and topotecan IC_50_ values for SW620 cells were approximately 4 μM and 21 nM, respectively. The GI_50_ values were similar to the IC_50_ values, namely 6 μM for cisplatin and 26 nM for topotecan (Fig. 1). On the other hand, dose–response curves obtained for SKOV3 cells barely reached the 50% reduction mark, resulting in a GI_50_ that was more than 2 times higher than the IC_50_ (17 μM vs 7 μM for cisplatin and 138 nM vs 68 nM for topotecan, respectively), indicating that there is a significant number of viable cells left even at the highest concentration of the drug (Fig. 1). These data suggest that the cytotoxic effects of cisplatin and topotecan in SKOV3 cells are less prominent than in SW620 cells.

The MTS assay data imply that the response to different drugs within one cell line (either SW620 or SKOV3) may be similar in terms of cell death or proliferation arrest. To investigate the processes potentially involved in drug-induced effects, we performed a pilot experiment with SW620 and SKOV3 cells treated with cisplatin and topotecan in concentrations corresponding to the IC_50_ values. We used different selective inhibitors to determine which type of cell death was induced by these compounds: 25 µM of Q-VD-Oph (“Q-VD”, pan-caspase-targeting apoptosis inhibitor), 12.5 µM of chloroquine (autophagy inhibitor), 20 µM of necrostatin-1 (RIPK1-targeting necroptosis inhibitor), and 2.5 µM of ferrostatin-1 (ferroptosis inhibitor). Q-VD was the only inhibitor able to partially restore the number of living SW620 cells after the treatment, which allowed us to assume that apoptosis provides the main contribution to cell death caused by cisplatin and topotecan (Supplementary Fig. 1a). Therefore, we used Q-VD for further study of cisplatin and topotecan action in the model cell lines. Furthermore, the lack of prominent cell number recovery observed in SKOV3 cells (Supplementary Fig. 1b) suggests that their cytotoxic response to cisplatin and topotecan may be weak, thus supporting our initial observations based on the GI_50_ values.

### Mechanisms of cisplatin action on SW620 cells

**Fig. 2:**
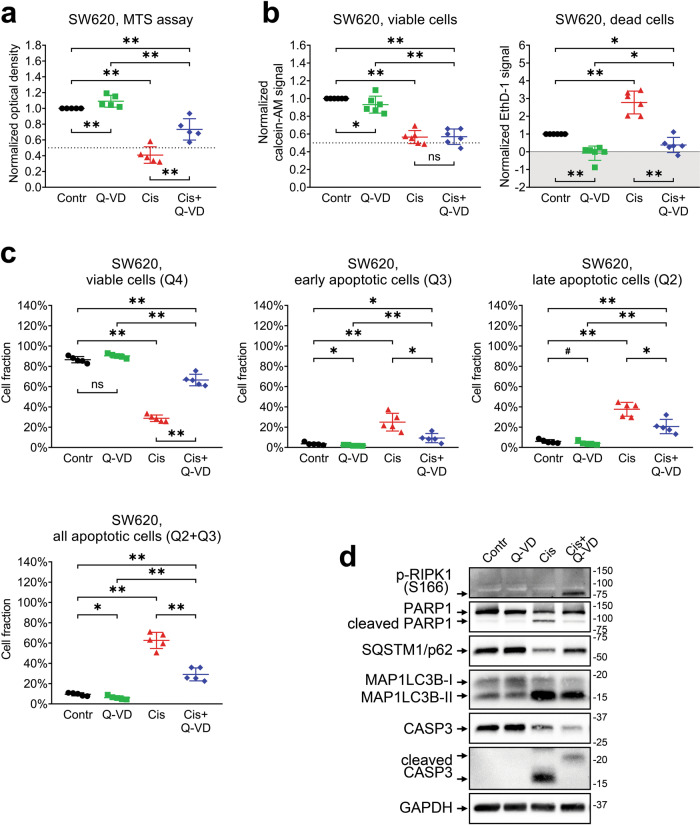
Different approaches used to evaluate SW620 cell viability after treatment with cisplatin and Q-VD. The cells were treated with 5 μM of cisplatin in the presence or absence of 25 μM of Q-VD. **a** Relative optical density values were obtained with the MTS assay. The data are normalized to the “Contr” sample; lines and whiskers indicate mean ± standard deviation (*N* = 5). The dashed line indicates a 50% reduction in the MTS signal. **b** Calcein-AM and EthD-1 signals were obtained via the LIVE/DEAD assay. The data are normalized to the “Contr” sample; lines and whiskers indicate mean ± standard deviation (*N* = 6). The dashed line indicates a 50% reduction in the calcein-AM signal, and the gray area indicates the EthD-1 signal that is below the average background level. **c** Fractions of viable, apoptotic, and necrotic cells estimated via Annexin V/PI assay. The lines and whiskers indicate mean ± standard deviation (*N* = 5). **d** Western blot analysis of protein levels. The samples are indicated above; proteins of interest are indicated on the left; molecular weight markers are indicated on the right. “Contr” – control sample; “Cis” – cisplatin.

Based on the results described above, we assumed that cisplatin has a cytotoxic effect on SW620 cells presumably exerted through apoptosis activation. To investigate this idea in a more detailed way, we treated the cells with 5 μM of cisplatin in the presence or absence of 25 μM Q-VD and compared cell viability data using MTS, LIVE/DEAD, Annexin V/PI, or Western blotting assays (Fig. 2, Supplementary Figs. 2 and 3).

In agreement with our pilot experiment (Supplementary Fig. 1), the MTS assay demonstrated a significant decrease in the viability of cisplatin-treated cells that was partially rescued upon the addition of Q-VD (see Fig. 2a). The LIVE/DEAD assay confirmed the reduction in the number of viable cells after cisplatin treatment and provided a direct confirmation of apoptosis indicated by significant increase in the number of dead cells, which was prevented by Q-VD (Fig. 2b). We also verified that the LIVE/DEAD assay provides consistent detection of living and dead SW620 cells via confocal microscopic imaging. The analysis confirmed that the calcein-AM and EthD-1 signals are mutually exclusive and correctly correspond to viable and dead cells (Supplementary Fig. 2).

Surprisingly, the addition of Q-VD to cisplatin did not result in significant recovery of the calcein-AM signal despite clear inhibition of cell death (Fig. 2b). This observation implies that cisplatin-induced decrease in SW620 viable cell numbers is to a large extent facilitated via proliferation arrest that does not result in actual cell death. This is also the first discrepancy we observed between the otherwise similar MTS and LIVE/DEAD assays.

We next evaluated the specific fractions of treated cells using flow cytometry with Annexin V/PI staining. It confirmed that cisplatin treatment induced apoptosis in SW620 cells and revealed that the majority of cisplatin-treated cells were in the state of early apoptosis (Fig. 2c, Supplementary Fig. 3). This fact is in agreement with our earlier suggestion that the cytostatic and stress-inducing actions of cisplatin are more prominent than its cytotoxic effect. Q-VD was able to rescue the cells from early apoptosis but not late apoptosis/secondary necrosis state.

Because cisplatin-induced cell death can involve various molecular mechanisms including apoptosis, necroptosis, and autophagy [23, 24] we evaluated the levels of several programmed cell death biomarkers and regulators using western blotting (Fig. 2d). Processing of caspase-3 and cleavage of poly(ADP-ribose) polymerase-1 (PARP1) are essential steps of apoptosis, RIPK1 phosphorylation is required for necroptosis, while SQSTM1/p62 degradation and MAP1LC3B lipidation are indicative of autophagy. Detection of cleaved forms of caspase-3 and PARP1 in cell lysates after incubation with cisplatin once again demonstrated activation of the apoptotic pathway in cisplatin-treated SW620 cells [25, 26].

Besides caspase-3 and PARP1 activation, western blot revealed that cisplatin treatment resulted in reduction of SQSTM1/p62 level and accumulation of MAP1LC3B-II (Fig. 2d), a standard autophagosome marker generated by the conjugation of cytosolic MAP1LC3B-I to phosphatidylethanolamine on the surface of nascent autophagosomes [27–30]. Simultaneous treatment with Q-VD and cisplatin also resulted in phosphorylation of RIPK1 kinase that may indicate the activation of pro-necroptotic machinery due to caspase inhibition. The fraction of cells in the state of early necroptosis (Q1 area, Fig. 2c) was negligible in all samples. However, inhibition of cisplatin-induced apoptosis with Q-VD still resulted in a significantly larger fraction of dead cells (Q2 area) than in the control sample, implying that some cells underwent necroptotic death.

The results described above demonstrate that cisplatin treatment of SW620 cells leads to both cell proliferation arrest and induction of apoptosis. While Q-VD treatment rescues cells from early apoptosis, cytostatic effects of cisplatin are not dependent on caspase activity. Therefore, it would be incorrect to interpret the cisplatin-induced reduction in the MTS assay signal as cytotoxic, cytostatic, or complex without more detailed investigation using additional methodological approaches.

### Topotecan exerts different effects on SW620 and SKOV3 cells

Because the sensitivity of different cell lines to cytotoxic and cytostatic effects of the same drug can vary, we sought to determine which details of the viability evaluation techniques should be given special attention in order to assess the effect of the drug correctly. To achieve this goal, we examined the response of SW620 and SKOV3 cells to treatment with the topoisomerase I inhibitor topotecan.

**Fig. 3:**
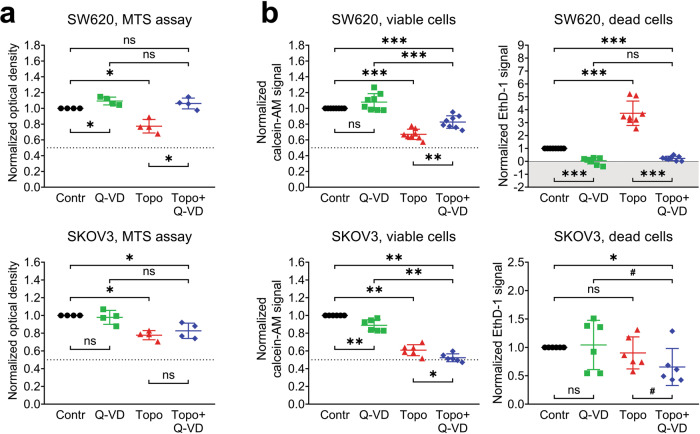
Cytotoxic and cytostatic effects of topotecan treatment in SW620 and SKOV3 cell lines (microplate-based assays). The cells were treated with topotecan (20 nM for SW620, 70 nM for SKOV3) in the presence or absence of 25 μM of Q-VD. **a** Relative optical density values obtained via the MTS assay. The data are normalized to the respective “Contr” samples; the lines and whiskers indicate mean ± standard deviation (*N* = 4). The dashed lines indicate a 50% reduction in the MTS signal. **b** Calcein-AM and EthD-1 signals were obtained via the LIVE/DEAD assay. The data are normalized to the respective “Contr” samples; the lines and whiskers indicate mean ± standard deviation (*N* = 8 for SW620, *N* = 6 for SKOV3). The dashed lines indicate a 50% reduction in the calcein-AM signal, and the gray area indicates the EthD-1 signal that is below the average background level. “Contr” – control samples; “Topo” – topotecan.

Similarly to the cisplatin treatment experiments described above, both the MTS and LIVE/DEAD assays demonstrated that incubation of SW620 and SKOV3 cells with topotecan at the IC_50_ concentration (20 nM for SW620 and 70 nM for SKOV3) significantly decreased the number of viable cells (Fig. 3a, b). However, it is remarkable that the addition of Q-VD to topotecan completely restored the MTS signal and partially restored the calcein-AM signal in SW620 cells, but had no effect on SKOV3 cells, suggesting that topotecan does not induce caspase-dependent apoptosis in SKOV3 cells. This idea is supported by comparison of the number of dead cells detected using EthD-1 staining: SW620 cells display a pattern similar to the one observed during cisplatin treatment (significant cell death induction by topotecan alone that is abolished by Q-VD addition), but there are no significant differences between the experimental groups of SKOV3 cells. This is strong evidence that SKOV3 cells have prominent resistance to the cytotoxic action of topotecan, and for this cell line, the topotecan-induced decrease in the MTS assay values should be considered a purely cytostatic effect without any relevant contribution to cell death.

**Fig. 4:**
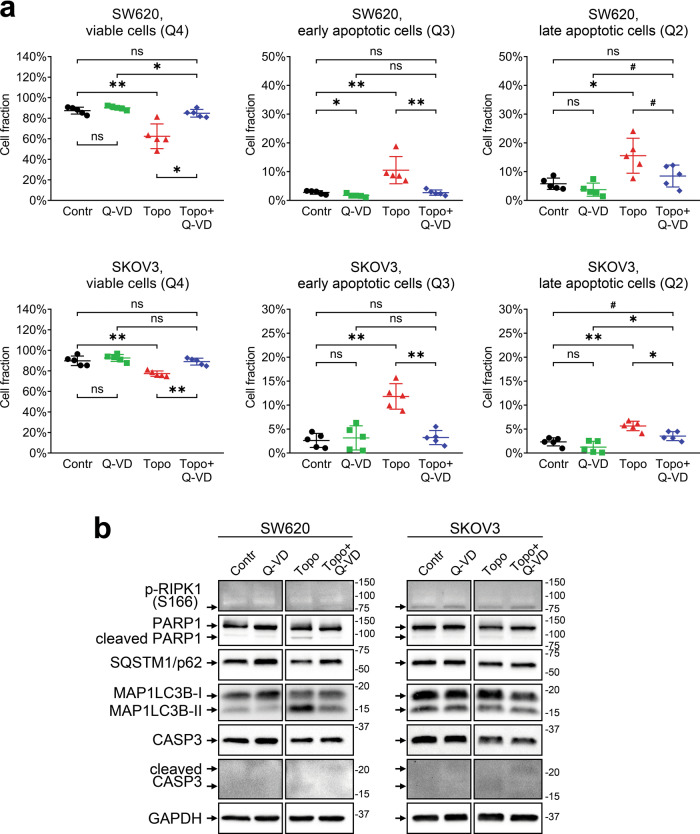
Cytotoxic effects of topotecan treatment in SW620 and SKOV3 cell lines (Annexin V/PI and Western blot assays). The cells were treated with topotecan (20 nM for SW620, 70 nM for SKOV3) in the presence or absence of 25 μM of Q-VD. **a** Fractions of viable and apoptotic cells estimated with the Annexin V/PI assay. The lines and whiskers indicate mean ± standard deviation (*N* = 5). **b** Western blot analysis of protein levels. The samples are indicated above; the proteins of interest are indicated on the left; molecular weight markers are indicated on the right. “Contr” – control samples; “Topo” – topotecan.

Annexin V/PI analysis confirmed our observations (Fig. 4a). Topotecan-treated SW620 cells displayed a significant decline in the fraction of living cells, while approximately 25% of cells were labeled as apoptotic (Q2 and Q3 areas). At the same time, the addition of Q-VD clearly restored the living cell fraction and decreased the content of apoptotically altered cells, as expected based on the MTS and LIVE/DEAD assay results.

Despite a significant reduction in the number of viable cells (Fig. 3a, b), topotecan-treated SKOV3 cells displayed very weak apoptosis-associated changes based on flow cytometry (Fig. 4a). Topotecan caused a marginal (about 13%) decrease in the relative content of living cells. The main contribution to the Annexin V-positive subpopulation of SKOV3 cells is provided by cells in early apoptosis (gate Q3), while the increase in the late apoptosis fraction is negligible (Fig. 4a). We confirmed the lack of pro-apoptotic changes in SKOV3 cells using western blotting and detected very low level of cleaved PARP1 and the lack of activated caspase-3 (Fig. 4b). Surprisingly, the level of processed caspase-3 was also very low in topotecan-treated SW620 cells. Because Q-VD was still able to prevent cell death, we suppose that topotecan may induce apoptosis in SW620 cells through activation of other caspases than caspase-3 or trigger fast degradation of its active form.

Similarly to cisplatin, topotecan promoted autophagy in SW620 cells, which was manifested in MAP1LC3B lipidation and a decrease in the SQSTM1/p62 level. However, there were no changes in RIPK1 phosphorylation. There were no consistent differences in autophagic or necroptotic proteins in SKOV3 cells upon topotecan treatment (Fig. 4b).

**Fig. 5:**
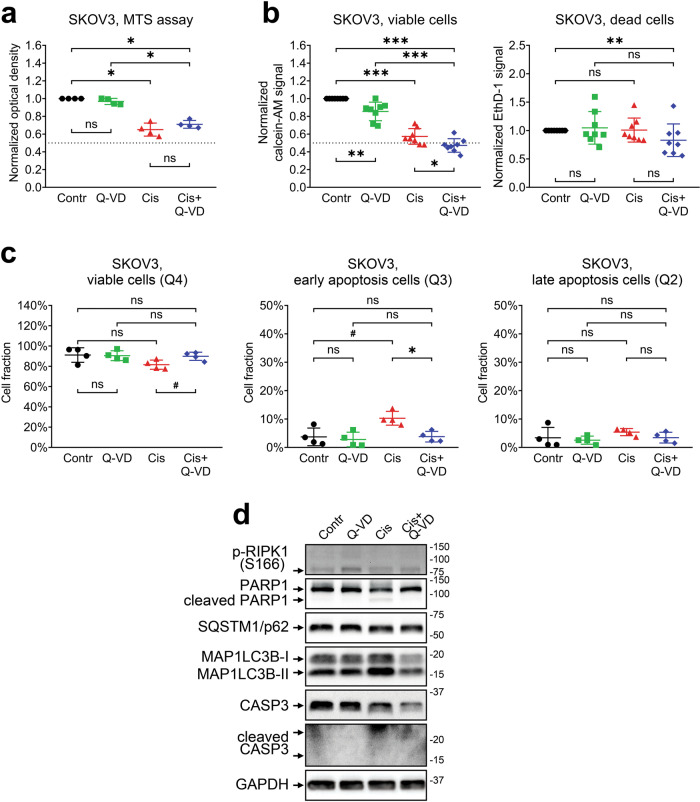
Cytotoxic and cytostatic effects of cisplatin treatment in SKOV3 cells. The cells were treated with 5 μM cisplatin in the presence or absence of 25 μM of Q-VD. **a** Relative optical density values obtained via the MTS assay. The data are normalized to the respective “Contr” sample; the lines and whiskers indicate mean ± standard deviation, *N* = 4. The dashed line indicates a 50% reduction in the MTS signal. **b** Calcein-AM and EthD-1 signals were obtained via the LIVE/DEAD assay. The data are normalized to “Contr” sample; the lines and whiskers indicate mean ± standard deviation, *N* = 8. The dashed line indicates a 50% reduction in the calcein-AM signal. **c** Fractions of viable and apoptotic cells estimated via the Annexin V/PI assay. The lines and whiskers indicate mean ± standard deviation, *N* = 4. **d** Western blot analysis of protein levels. The samples are indicated above; the proteins of interest are indicated on the left; molecular weight markers are indicated on the right. “Contr” – control samples; “Cis” – cisplatin.

Because SKOV3 cells were much more resistant to the cytotoxic action of topotecan, we decided to check their sensitivity to cisplatin-based cell death induction using our established set of experiments. As expected, SKOV3 cells were almost completely insensitive to the cytotoxic effects of cisplatin, so the reduction in the number of viable cells after cisplatin treatment was due to proliferation arrest only (Fig. 5). This fact is in complete agreement with our earlier observation that even the highest concentration of cisplatin could not reduce the number of viable SKOV3 cells below 40% of the control level due to the lack of cell death (Fig. 1). In addition, cisplatin treatment of SKOV3 cells was unable to induce prominent autophagy- and necroptosis-associated protein changes previously observed in SW620 cells (Figs. 2d and 5d). Therefore, we conclude that SKOV3 cells, while displaying susceptibility to the cytostatic action of various drugs, may exhibit universal resistance to the cytotoxic stimuli.

## Discussion

The action of the majority of drugs may be represented as a combination of two processes: activation of cell death (cytotoxic effect) and inhibition of proliferation and metabolism (cytostatic effect). Therefore, a competent researcher should clearly understand which methods should be used to discern between these two while estimating the effect of a drug.

In this study, we evaluated the different approaches to assess cell viability and cell death for two drugs with different mechanisms of action, cisplatin and topotecan, using two cell lines with a different p53 mutation status. These drugs induce DNA damage via different mechanisms: topotecan binds to the topoisomerase I–DNA complex, leading to double-stranded DNA breaks during DNA replication [31, 32], whereas the main mechanism of platinum-based agents is covalent binding to the DNA bases themselves and the formation of DNA cross-links [33, 34]. In both cases, prolonged DNA damage induction should result in proliferation arrest and cell death induction; however, p53 mutations can render cancer cells partially or completely insensitive to DNA damage–associated cell stimuli.

The reduction in the MTS signal obtained from the cells is often referred to as “decrease in cell viability”. However, this term is unclear and it sometimes may confuse the reader into thinking that the cells of interest are undergoing cell death without providing direct evidence of such event. We found that both the SW620 and SKOV3 cell lines respond to cisplatin and topotecan treatment with a significant reduction in the MTS assay and calcein-AM staining signals. Therefore, it may be tempting to assume that the observed processes would be similar. However, our results clearly indicate that apoptotic death is only induced in SW620 cells, while the vast majority of SKOV3 cells remain viable. In that case, the statement about a “decrease in cell viability” would be misleading in regard of SKOV3 cells and should instead be rephrased as a “decrease in the number of viable cells”. Moreover, statements about cell viability and cell death induction should never be made based on the MTS assay alone; they should be always supported by data obtained using additional experimental approaches capable of direct or indirect detection of dying cells. The advantages and limitations of such experimental approaches are discussed below.

Cisplatin and topotecan exert both cytostatic and cytotoxic actions upon SW620 cells. According to the MTS assay, the cisplatin-induced decrease in the number of viable SW620 cells was restored by addition of the pan-caspase inhibitor Q-VD, suggesting that drug treatment mainly results in apoptosis induction (Fig. 2a). This conclusion is supported by high fraction of Annexin V–positive cells and processing of capsase-3 and respective cleavage of PARP1 in cisplatin-treated cells (Fig. 2c, d). At the same time, the LIVE/DEAD assay detected no recovery of the number of living cells in samples treated with cisplatin and Q-VD combination despite complete inhibition of cell death (Fig. 2b). A possible explanation for this discrepancy is that the MTS and LIVE/DEAD assays utilize different enzymes to assess cell metabolic activity. It is worth noting that cisplatin-treated SW620 cells displayed a 60% reduction in the MTS signal, but only a 45% reduction in the calcein-AM signal (Fig. 2a, b). Cisplatin treatment can affect mitochondria [35–37] and, therefore, cause an additional decrease in the MTS signal compared to the calcein-AM signal obtained in the same conditions. In this case, partial recovery of the MTS assay signal in the cells treated by combination of cisplatin and Q-VD may reflect the recovery of cell mitochondria from stress caused by cisplatin-induced caspase activation. It is critical to check in advance if the compounds of interest could affect MTS or other enzyme-based viability assay in any other way besides modulating the number of viable cells.

While the LIVE/DEAD assay revealed a significant increase in the EthD-1 signal in cisplatin- and topotecan-treated SW620 cells, Annexin V/PI analysis demonstrated that completely dead or late apoptotic cells constitute less than 25% of the whole cell population (Figs. 2–4). It is likely that some metabolic enzymes retain their activity during the initial apoptosis induction, so the cells detected by the MTS assay and calcein-AM dye as viable may actually include a fraction of cells in the state of early apoptosis, which is reversible [38] and may be recovered by Q-VD. This idea is consistent with flow cytometry data, according to which the majority of SW620 cells treated with cisplatin go into early apoptosis and most likely exhibit proliferation arrest due to prolonged stress. It also explains why Q-VD does not provide recovery of the calcein-AM signal; the fraction of rescued late apoptotic/necrotic cells is too small to cause significant changes, and some cells may undergo RIPK1-dependent necroptotic cell death even in caspase-inactivating conditions (Fig. 2c, d). On the other hand, topotecan treatment results in comparable fractions of early and late apoptotic SW620 cells, so the addition of Q-VD is accompanied by significant rescue of viable cell signals obtained via both the MTS and LIVE/DEAD assays.

There is another discrepancy observed between the data on Q-VD cell rescue obtained by LIVE/DEAD and AnnexinV/PI assays. EthD-1 staining indicates that Q-VD reduces the signal from dead cells to background level (Fig. 2b), while flow cytometry results display no change in late apoptotic cell fraction (Fig. 2c). There could be several factors explaining that. LIVE/DEAD staining is performed on attached cells and puts weak additional stress upon them, while flow cytometry assay includes trypsinization and several staining/washing steps that may promote terminal apoptosis/necrosis in the sample. There are differences between EthD-1 and PI properties (based on their molecular structure) and detection methods (evaluation of each single particle by flow cytometry versus detection of total signal from the whole well in LIVE/DEAD assay), and they could affect the outcome. The gating strategy in flow cytometry assay also has a significant impact on cell fraction estimation. All these details should be taken into account when drawing the conclusion from the experimental data.

We confirmed apoptotic death of SW620 cells by detecting a marked increase in the levels of cleaved caspase-3 and the 89 kD catalytic fragment of PARP1, two classic hallmarks of apoptosis [39, 40]. Q-VD prevents caspase-3 and PARP1 cleavage, abrogating apoptosis. However, the combination of cisplatin with Q-VD promotes phosphorylation of RIPK1, indicating the necroptotic shift in cell death signaling, a phenomenon described in the literature as a way to accomplish cell death in the condition of caspase deficiency [41, 42]. While we did not observe significant changes in the necroptotic cell fraction (Q1 area), conventional flow cytometry analysis cannot reliably determine whether Annexin V- and PI-positive cells in the Q2 area died through apoptosis or necroptosis. Researchers interested in precisely discerning between apoptosis and necroptosis may consider using imaging flow cytometry that can also analyze cell morphology, which is very different between apoptotic and necroptotic cells [43]. Moreover, we observed that cisplatin and topotecan increased the conversion of MAP1LC3B-I to MAP1LC3B-II and reduced the SQSTM1/p62 level in SW620 cells, indicating the autophagy activation. Autophagy is a dualistic process that may promote either cell death or cell resistance to stressful stimuli, including chemotherapy [44]. It is therefore important to evaluate possible autophagy-associated effects of tested drugs as they may modulate the response to the treatment or be associated with a mechanism of action [45]. For example, topotecan did not induce any consistent changes in autophagy markers, which may be due to its high specificity toward topoisomerase I, whereas cisplatin exerts a very general DNA-damaging effect that may be accompanied by global cellular processes like autophagy.

While the treatment response of SW620 cells included both cytostatic and cytotoxic components, SKOV3 cells failed to display any substantial cell death induction. This fact is consistent with the published data reporting SKOV3 resistance to cisplatin [46, 47] and is also very important for the correct understanding of principles behind drug screening studies. Because the efficacy of nonselective anticancer drugs (DNA alkylating agents, nucleoside analogs, and anti-microtubule agents) is directly related to their cytotoxicity [48], compounds without cytotoxic action should be recognized and excluded from the study as early as possible. Based on our data, we propose that comparison of IC_50_ and GI_50_ values can provide important initial information for this task [49]. Figure 1 illustrates that the GI_50_ for treatment-resistant SKOV3 cells is significantly higher than the IC_50_, while sensitive SW620 cells, display very similar IC_50_ and GI_50_. So, if the primary assessment of drug action by MTS assay results in GI_50_ value that is much higher than IC_50_, the researcher may assume that the drug of interest exerts cytostatic action, while the cytotoxic effect might be weak. Nevertheless, this suggestion should be confirmed by other methods.

Here we have demonstrated that comprehensive analysis of cell death induced by drug treatment of cancer cells is a very complex task that can only be achieved by combining several methodological approaches. On the other hand, certain specific questions can be properly addressed through the application of the most suitable approach. Thus, the applicability of different methods described above to the different areas of cell death investigation are summarized in Table 1 and discussed below.

**Table 1:** Advantages and disadvantages of methods used for drug cytotoxicity analysis.

Method	Advantages	Disadvantages	Application
MTT, MTS, XTT, WST-1	Inexpensive; Highly accessible; Relatively fast; Simple in execution.	Does not detect dead cells; Cells are analyzed in bulk; Additional steps in protocol may increase data variability (in case of MTT); Toxic to cells.	High-throughput screening
LIVE/DEAD assay	Allows to evaluate numbers of both viable and dead cells; Rapid process; Simple in execution; Moderately accessible; Can be optimized for microscopic studies.	Expensive; Cells are analyzed in bulk; Requires optimization for each cell line; Limited sensitivity in adherent cells [52].	Screening of small compound collections
Annexin V flow cytometry analysis	Estimates the fractions of viable cells, cells in early apoptosis, and cells in late apoptosis/necrosis; Analyzes individual cells.	Provides no information on total cell number; Does not discern between cells in late apoptosis and necrosis. Analysis of adherent cells requires additional procedures that may affect the viability; Cannot be scaled for high-throughput analysis.	Study of apoptosis in a limited number of samples; Identification of drug-sensitive and drug-resistant subpopulations.
Western blotting	Allows to estimate molecular mechanisms of drug action; High sensitivity.	Provides no information on cell numbers; Cells are analyzed in bulk; Requires high-quality antibodies against cell death markers; Cannot be scaled for high-throughput analysis.	Study of molecular mechanisms of drug action in a small number of samples.

Formazan-based assays (MTT, MTS, XTT, and WST-1) are suitable for massive high-throughput compound screening [50, 51] because they only require optical absorbance plate readers for the measurements, and the sample preparation is relatively fast and easy. However, these assays are unable to detect dead cells directly; instead, they can only be used for the exclusion of compounds that do not reduce the number of viable cells and not for the identification of truly cytotoxic drugs.

The LIVE/DEAD viability/cytotoxicity assay is similar to the MTT assay in many aspects, namely cells may be stained in multi-well plates without prior detachment from the surface, and the signals from a large number of samples can be measured in parallel. The major drawbacks include the necessity for a fluorescent plate reader and the relatively high price of the fluorescent dyes, which make the LIVE/DEAD assay too expensive for massive drug screenings. It is more applicable for identifying truly cytotoxic drugs in smaller libraries of drugs pre-selected using the MTT assay.

The flow cytometry-based Annexin V/PI assay represents a markedly different approach. It is used to analyze individual cells and to evaluate the ratios between viable and apoptotic/necrotic cells, but it does not allow assessing the total number of cells. Investigators should also consider that flow analysis of adherent cells requires a cell detachment step that could result in additional stress or even membrane damage. The sample preparation procedure is lengthy, and flow cytometers usually process the samples in succession; thus, this method is only efficient for analyzing a small number of samples.

Western blotting is the common method of choice in fundamental studies focused on investigating molecular mechanisms of drug action [16]. However, the procedure is time-consuming, requires a lot of different laboratory hardware and multiple specific antibodies, and includes multiple steps that may potentially affect the reproducibility and reliability of the method. It also cannot be scaled up to process a large number of samples with sufficient efficiency. Due to these limitations, western blotting is mostly used for very specific tasks directed at the detailed characterization of selected drugs and model cell lines.

Taken together, the widely used MTT assay or its analogs may demonstrate similar results for different drugs and/or cell lines despite distinct molecular mechanisms. Thus, SW620 and SKOV3 cells had a comparable IC_50_ for cisplatin—the main characteristic estimated with the MTT assay. However, an application of various techniques sheds light on the difference between cell lines. First, cisplatin was able to trigger apoptosis in SW620 cells but not in SKOV3 cells, inducing cytostatic as well as cytotoxic effects. Second, autophagy and necroptosis stimulated by this drug might modulate resistance in SW620 compared with SKOV3 cells. Third, inhibition of caspase activation may cause MTT signal increase, but does not necessarily result in the recovery of living cell numbers. All of these differences may play an important role in drug development, selection, and application in vivo.

## Conclusion

Drug toxicity is a key problem in pharmacology and is one of the main reasons for the high cost of drug development. While many studies characterize drug effects using just one or two methodological approaches, there is always a risk to form incorrect conclusions. It is important to keep in mind that the action of the drug includes its ability to induce cell death, inhibit metabolism, and/or block proliferation. By using this methodological analysis, we aim to provide a note for researchers in the field, advising them to choose the most suitable technique(s) to test their compounds/hypotheses and to draw accurate and consistent conclusions.

## Materials and methods

### Cell culture and treatments

The colorectal adenocarcinoma cell line SW620 (ATCC, CCL-227) and the ovarian adenocarcinoma cell line SKOV3 (ATCC, HTB-77) were kindly provided by the Department of Toxicology, Karolinska Institute (Stockholm, Sweden). The cells were cultured in Dulbecco’s Modified Eagle Medium (DMEM) with 4.5 g/L glucose (Gibco, Waltham, MA, USA) supplemented with antibiotic-antimycotic penicillin (100 U/mL) plus streptomycin (100 µg/mL) (Gibco), 1 mM sodium pyruvate (PanEco, Moscow, Russia), and 10% fetal bovine serum (Gibco). Cells were grown in a CO_2_ incubator (5% CO_2_) at 37 °C and split every 2–3 days using 0.15% trypsin solution (Gibco). Cells at the logarithmic growth phase were used for the experiments.

Throughout the experiments, the cells were stimulated with cisplatin (Teva, Tel Aviv, Israel) and topotecan (Merck, Darmstadt, Germany) for 72 h. To determine the type of cell death induced by these compounds, the cells were treated in presence of selective inhibitors of apoptosis (Q-VD-Oph, Selleck Chemicals LLC, Houston, TX, USA), autophagy (chloroquine, “CQ,” Sigma-Aldrich, Saint Louis, MO, USA), necroptosis (necrostatin-1, “Nec-1,” Abcam, Cambridge, UK), and ferroptosis (ferrostatin-1, “Fer-1,” Sigma-Aldrich). These inhibitors were added for 1 h prior to cisplatin or topotecan and kept for the entire treatment duration.

### MTS assay

Cells were plated into flat-bottomed 96-well plates (Nunc, Roskilde, Denmark), 1500 cells per well, and cultured in full growth medium overnight. The medium was then removed and replaced with 0.1 mL of fresh DMEM containing cisplatin or topotecan in the presence or absence of Q-VD, CQ, Nec-1, or Fer-1, as described above. The plates were incubated at 37 °C for 72 h. After the treatment, 20 µL of MTS (CellTiter 96 AQueous One Solution Cell Proliferation Assay, Promega, Madison, WI, USA) labeling reagent was added to each well, and the plates were incubated at 37 °C for another 3 h. Following MTS incubation, the spectrophotometric absorbance of the samples was detected by using a VarioScan Flash microplate reader (Thermo Fisher Scientific, Waltham, MA, USA) at 480 nm with a reference wavelength of 630 nm.

### LIVE/DEAD assay for mammalian cells

Cells were plated into flat-bottom 96-well plates (Nunc), 1500 cells per well, or glass-bottom 24-well Sensoplate plates (Greiner Bio-One, Kremsmünster, Austria), 5000 cells per well, and cultured in full growth medium overnight. The next day, the cells were treated with cisplatin, topotecan, and Q-VD for 72 h as described above. After the treatment, the cells were stained with LIVE/DEAD Viability/Cytotoxicity Kit for mammalian cells (Thermo Fisher Scientific) by diluting calcein-AM and ethidium homodimer-1 (EthD-1) in Dulbecco’s phosphate-buffered saline (DPBS, PanEco) and adding it to the wells. The final concentrations were 2 μM for calcein-AM and 4 μM for EthD-1.

Samples in 96-well plates were used for quantitative evaluation of viable and dead cell numbers using a VarioScan Flash microplate reader. The Calcein-AM signal from viable cells was detected 15 min after dye addition using an excitation filter at 485 nm and an emission filter at 517 nm. The EthD-1 signal from dead cells was detected 45 min after dye addition using an excitation filter at 530 nm and an emission filter at 617 nm. Wells with no cells were used for background signal evaluation, cells treated with 70% ethanol for 30 min were used as a positive control for EthD-1 staining.

Samples in 24-well Sensoplate plates were used to confirm the specificity of calcein-AM and EthD-1 staining using confocal microscopy. Images were taken using a Zeiss LSM 780 confocal microscope and ZEN 2010 software (Zeiss, Oberkochen, Germany). The calcein-AM signal from viable cells was detected 15 min after dye addition using an excitation wavelength of 488 nm. The EthD-1 signal from dead cells was detected 45 min after dye addition using excitation wavelength of 561 nm. The images were processed using ZEN 3.2 Blue Edition software (Zeiss).

### Western blotting

The cells were harvested using 0.1% trypsin-EDTA solution (Gibco). Next, the cells were centrifuged (1000 rcf, 4 min, +4 °C), washed with DPBS (PanEco), centrifuged again (1 000 rcf, 4 min, +4 °C), and the pellet was lysed in radioimmunoprecipitation assay (RIPA) buffer (25 mM Tris-HCl, 150 mM NaCl, 1% NP-40, 0.5% sodium deoxycholate) containing Halt protease inhibitor cocktail (Thermo Fisher Scientific) and phosphatase inhibitor cocktail (Sigma-Aldrich) for 15 min on ice. After centrifugation (15 000 rcf, 15 min, +4 °C), a part of the supernatant was taken to determine the protein concentration, and another part was used for western blotting. The protein concentration was assessed using a BCA Protein Assay Kit (Thermo Fisher Scientific). Protein samples were mixed with a Laemmli buffer containing 150 mM dithiothreitol and incubated at 95 °C for 5 min. Protein was separated using sodium dodecyl sulfate–polyacrylamide gel electrophoresis (SDS-PAGE). Protein was transferred to a nitrocellulose membrane at 110 V for 110 min in a wet tank transfer system (Bio-Rad, Hercules, CA, USA). Then, the membranes were incubated in a 5% solution of nonfat milk powder in TBST buffer (20 mM Tris, 150 mM NaCl, 0.025% Tween 20, pH 7.4) for 45 min at room temperature to block nonspecific protein binding sites. After that, the membranes were washed in TBST (4 × 5 min) and incubated with primary antibody at +4 °C for approximately 16 h. The membranes were washed in TBST (4 × 5 min), and secondary antibody was added in 2.5% nonfat milk powder solution and incubated for an hour at room temperature. The membranes were washed (4 × 5 min) in TBST, and a signal was induced using Clarity Western ECL substrate (Bio-Rad). Chemiluminescence detection and image analysis were performed using the ChemiDoc XRS^+^ System (Bio-Rad). The following primary antibodies were used at indicated dilution: rabbit monoclonal to phospho-RIPK1 (Ser166, clone D1L3S) diluted 1:1 000 (65746, Cell Signaling Technology, Danvers, MA, USA); rabbit polyclonal to PARP1 diluted 1:1 000 (ab137653, Abcam); mouse monoclonal to SQSTM1/p62 (clone 2C11) diluted 1:1 000 (ab56416, Abcam); rabbit polyclonal to MAP1LC3B diluted 1:3 000 (ab51520, Abcam); mouse monoclonal to CASP3 (clone 19/Caspase-3/CPP32) diluted 1:1 000 (610323, BD Biosciences, San Diego, CA, USA); rabbit polyclonal to cleaved CASP3 diluted 1:1 000 (9661, Cell Signaling Technology); rabbit monoclonal to GAPDH (clone 14C10) diluted 1:3 000 (2118, Cell Signaling Technology). The original uncropped images of western blot membranes are provided in Supplementary Data.

### Fluorescence-activated cell sorting (FACS) analysis

The FITC Annexin V Apoptosis Detection Kit I (BD Biosciences) was used to detect the level of apoptotic and necrotic cells. The cells were harvested using 0.15% trypsin solution and counted using the Z1 Particle Counter (Beckman Coulter, Chaska, MN, USA). A total of 1 × 10^5^ cells were taken for analysis, centrifuged (500 rcf, 4 min, +4 °C), washed with DPBS, and centrifuged again (500 rcf, 4 min, +4 °C). The cell pellet was resuspended in 100 µL Annexin-binding buffer (BD Biosciences). Next, 2 µL of Annexin V-FITC (BD Biosciences) was added, and the cells were incubated with Annexin V for 15 min in the dark at room temperature, and then placed on ice. Immediately before measurement, propidium iodide (PI) was added to the samples to a final concentration of 0.5 µg/mL, and the samples were analyzed with the BD FACSCanto II cell analyzer (BD Biosciences). Flow cytometry data were processed using FlowJo v10.7 software (BD Biosciences).

### Data processing and statistical analysis

Three or more independent repeats were performed for each experiment unless stated otherwise in the figure legend. Calculations of the half maximal inhibitory concentration (IC_50_) and the half maximal growth inhibitory concentration (GI_50_), statistical analysis, and data plotting were performed using OriginPro 2021 software (OriginLab Corporation, Northampton, MA, USA) and GraphPad Prism 8 software (GraphPad Software, San Diego, CA, USA). The differences between experimental groups were analyzed using two-sided Mann–Whitney U-test (ns, non-significant difference; *p < 0.05; **p < 0.01; ***p < 0.001).

## Supplementary information

Supplementary data

Original Data File

## Data Availability

The authors have no data to deposit in a repository.
