# Supplementary material for: Drug toxicity assessment: cell proliferation versus cell death
Source: Cell Death Discov. 2022 Oct 14;8:417. doi: 10.1038/s41420-022-01207-x (PMC9568594; doi:10.1038/s41420-022-01207-x)
Supplement: Supplementary file 1 — Supplementary data [file 41420_2022_1207_MOESM1_ESM.pdf]

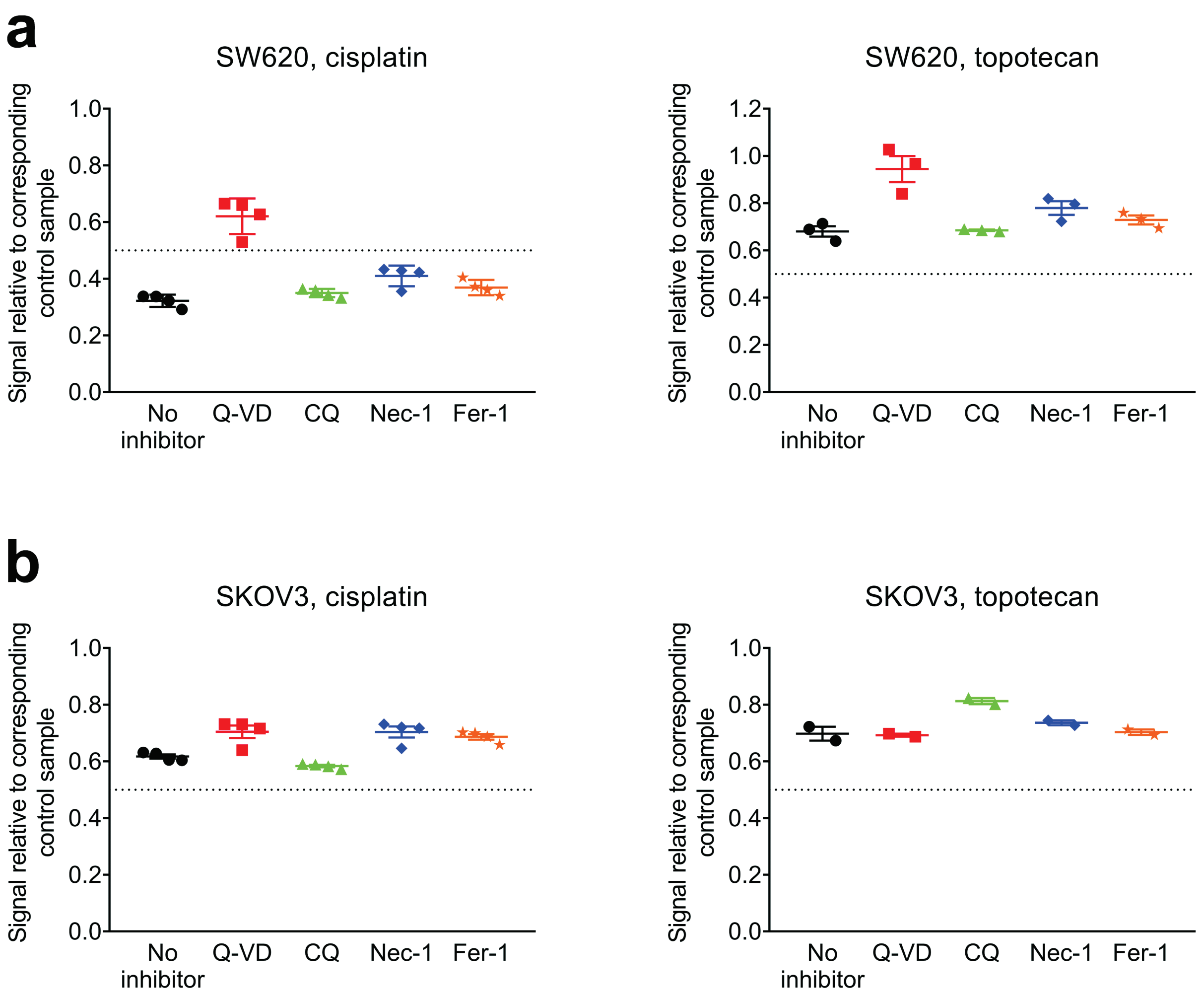

Supplementary Figure 1. Rescue of viable cell numbers after cisplatin or topotecan treatment in combination with cell death inhibitors. The cells were treated with cisplatin (5 µM for both cell lines) or topotecan (20 nM for SW620, 70 nM for SKOV3) in the combination with 25 µM Q-VD, 12.5 µM chloroquine (CQ), 20 µM necrostatin-1 (Nec-1), or 2.5 µM ferrostatin-1 (Fer-1). The MTS assay signal values obtained from SW620 (**a**) or SKOV3 (**b**) cells were normalized to the values obtained from corresponding samples treated with cell death inhibitors only to reflect the relative signal reduction. The lines and whiskers indicate mean ± standard error of the mean, N = 4 for cisplatin experiments, N = 3 for SW620 treated with topotecan, N = 2 for SKOV3 treated with topotecan (technical replicates).

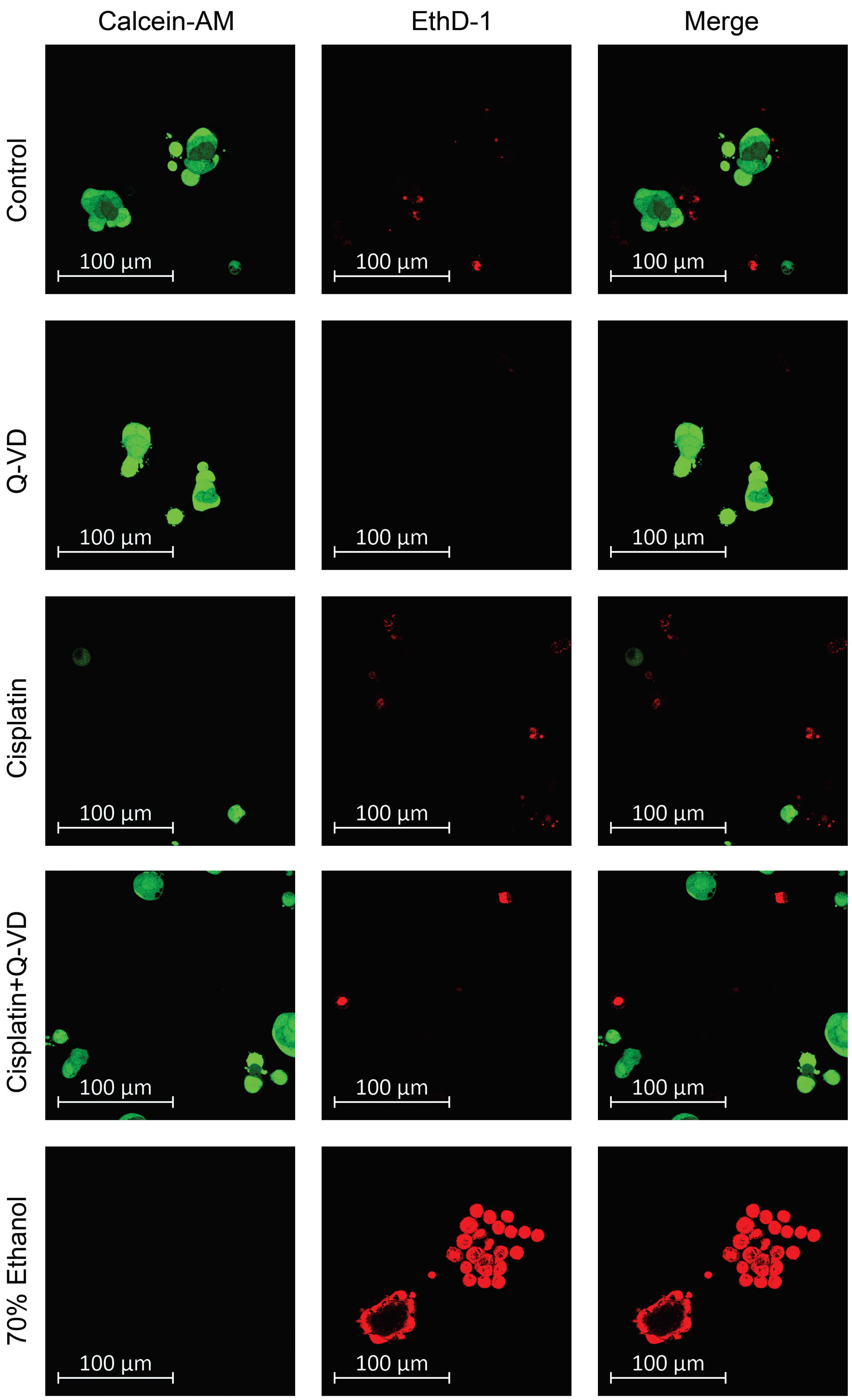

Supplementary Figure 2. Mutually exclusive staining of viable and dead SW620 cells by LIVE/DEAD assay kit. The cells were treated with 5 μM cisplatin and 25 μM of Q-VD or fixed with 70% ethanol and then stained with the LIVE/DEAD kit. The representative images were taken to demonstrate that green calcein-AM dye selectively stains the viable cells, while red EthD-1 dye is only specific to the nuclei of the dead cells.

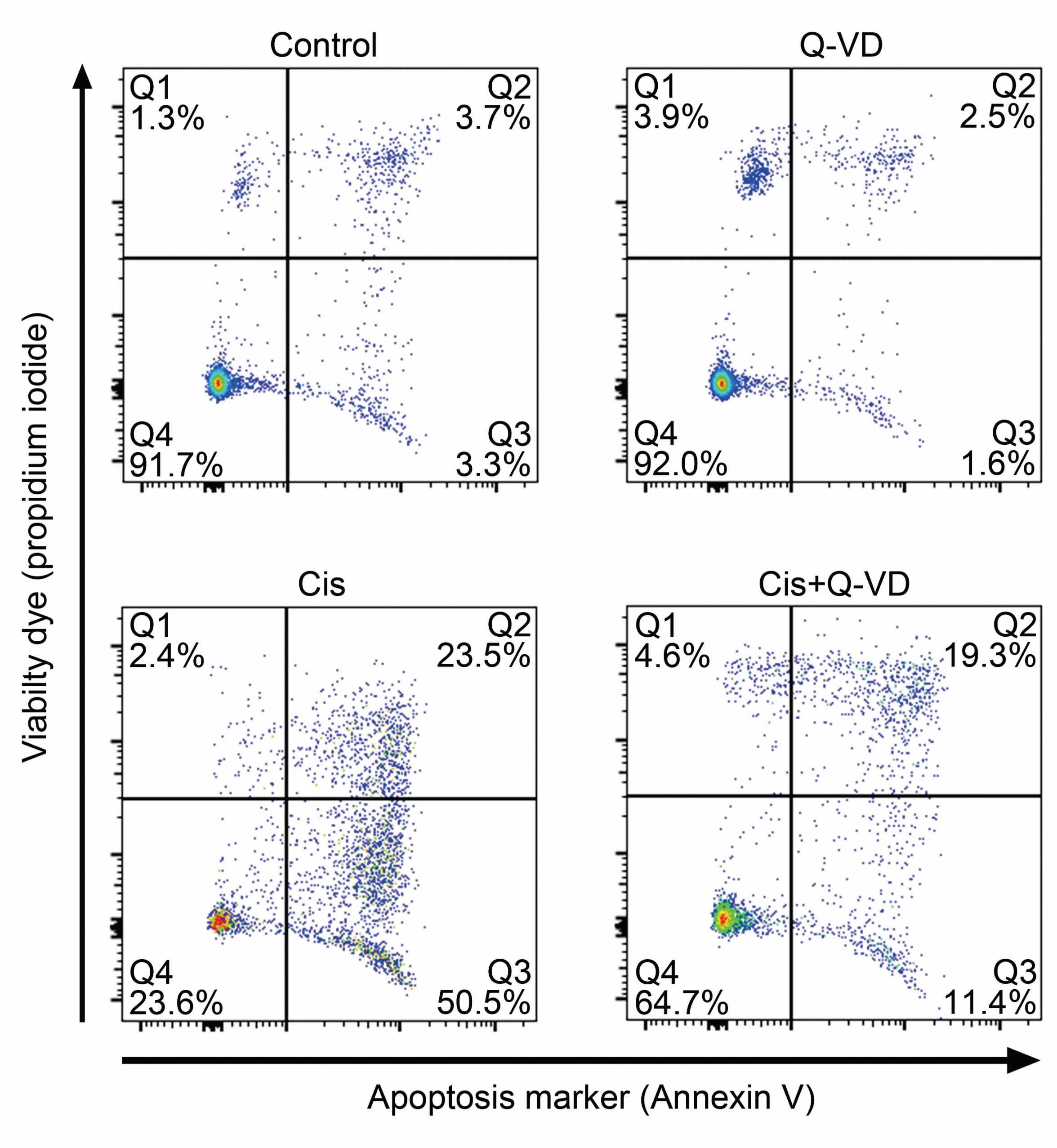

Supplementary Figure 3. Scatter plot representation of the Annexin V/PI assay results obtained for cisplatin- and Q-VD-treated SW620 cells (Figure 2c). The numbers correspond to early necrotic (Q1), late apoptotic or completely dead (Q2), early apoptotic (Q3), and viable (Q4) cell fractions. “Contr” – control sample; “Cis” – cisplatin.
