# Supplementary material for: Drug toxicity assessment: cell proliferation versus cell death
Source: Cell Death Discov. 2022 Oct 14;8:417. doi: 10.1038/s41420-022-01207-x (PMC9568594; doi:10.1038/s41420-022-01207-x)
Supplement: Supplementary file 2 — Original Data File [file 41420_2022_1207_MOESM2_ESM.pdf]

Figure 2e:

p-RIPK1 (S166)

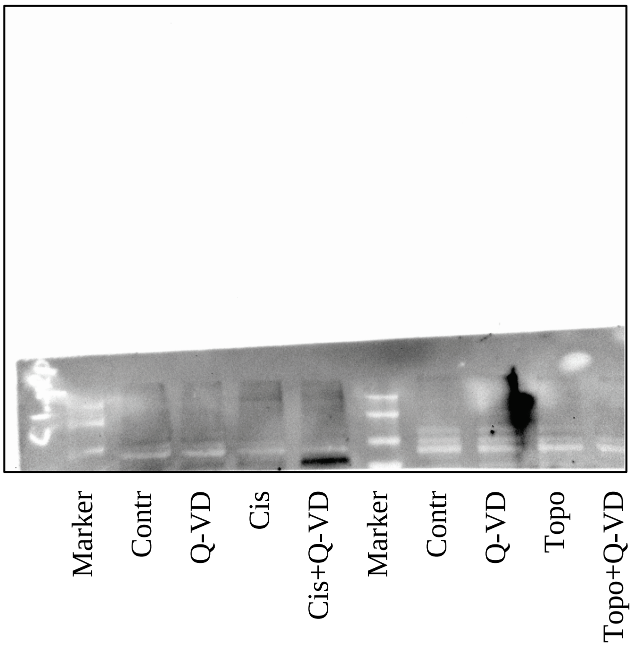

PARP1

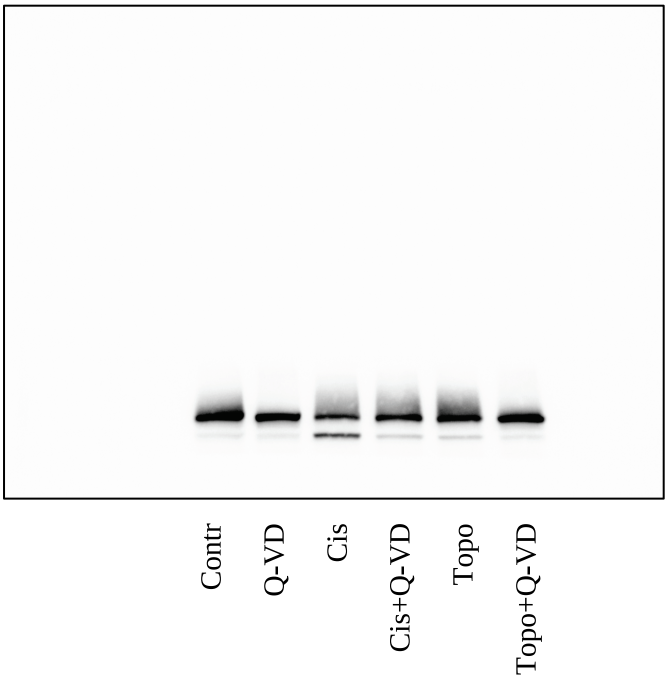

SQSTM1/p62

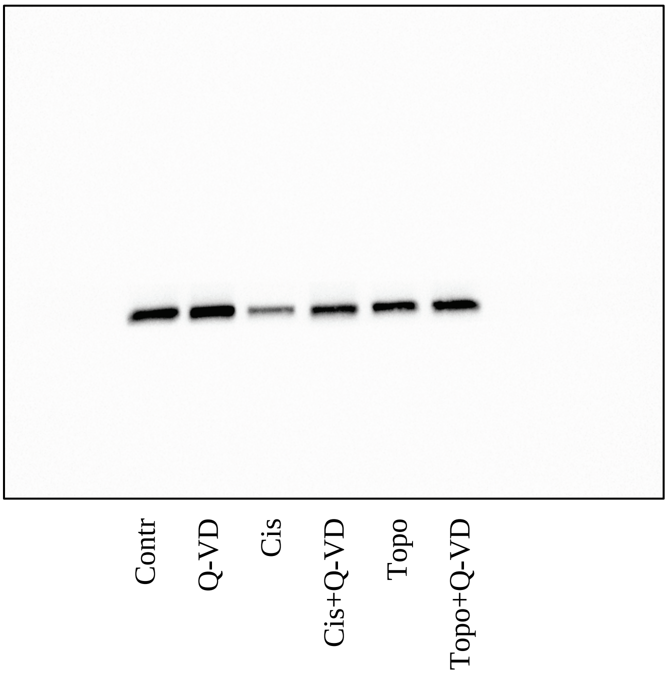

MAP1LC3B

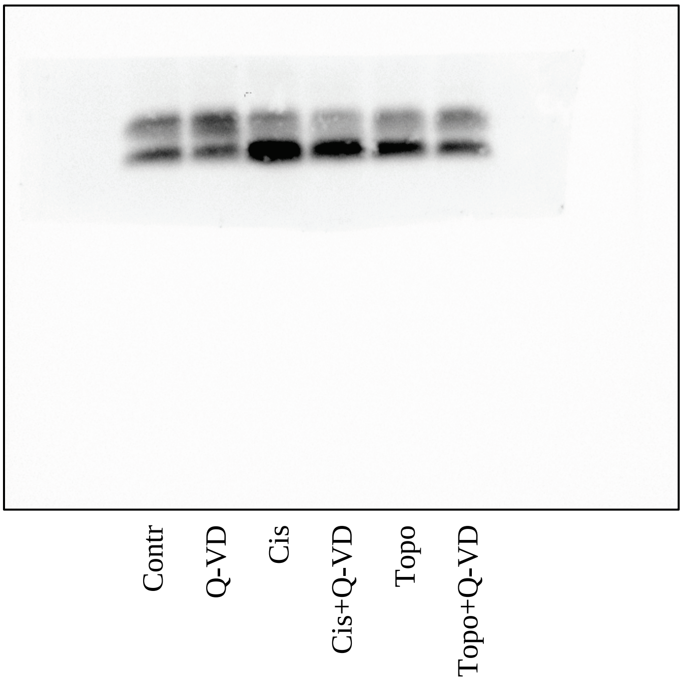

CASP3

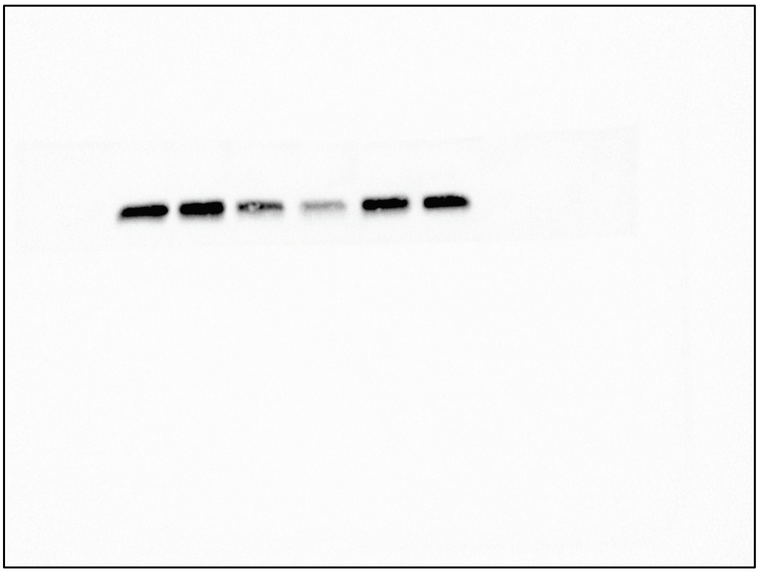

Contr
Q-VD
Cis
Cis+Q-VD
Topo
Topo+Q-VD

Cleaved CASP3

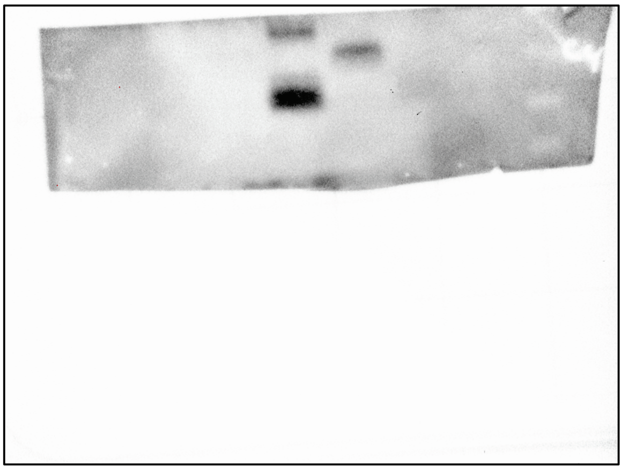

Contr
Q-VD
Cis
Cis+Q-VD
Topo
Topo+Q-VD

GAPDH

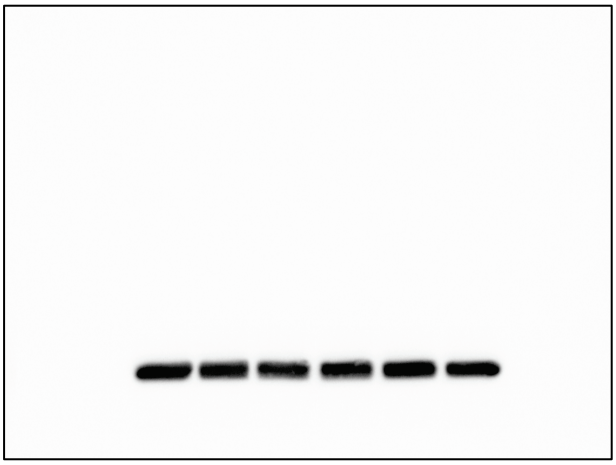

Figure 3d – SW620:

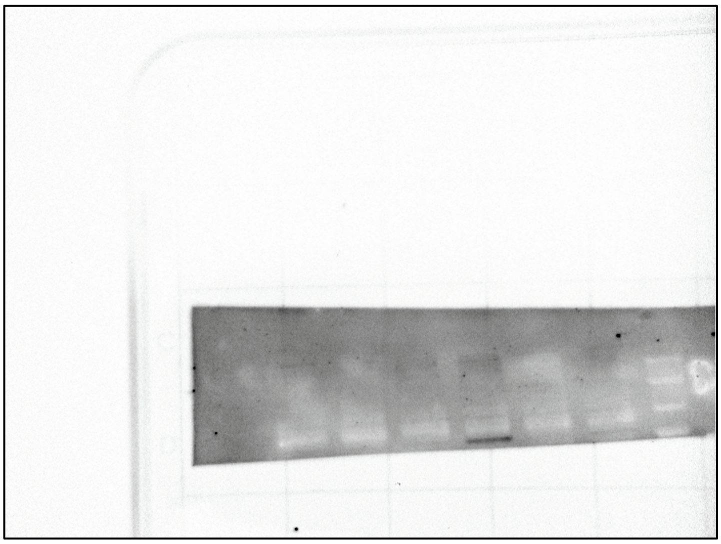

p-RIPK1 (S166)

Contr Q-VD Cis Cis+Q-VD Topo Topo+Q-VD

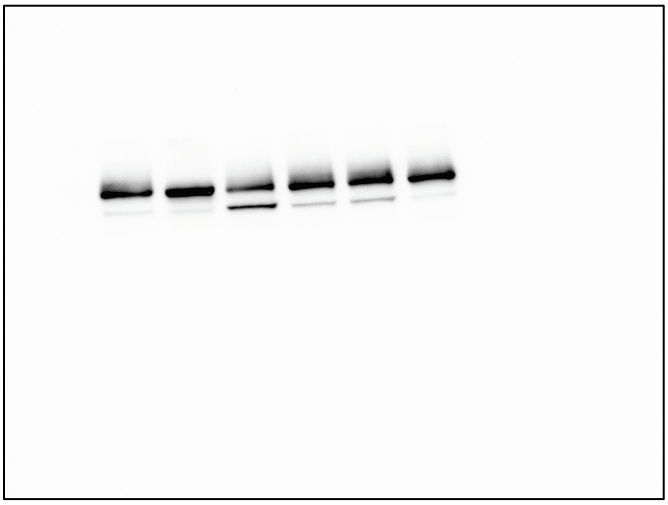

PARP1

Contr Q-VD Cis Cis+Q-VD Topo Topo+Q-VD

SQSTM1/p62

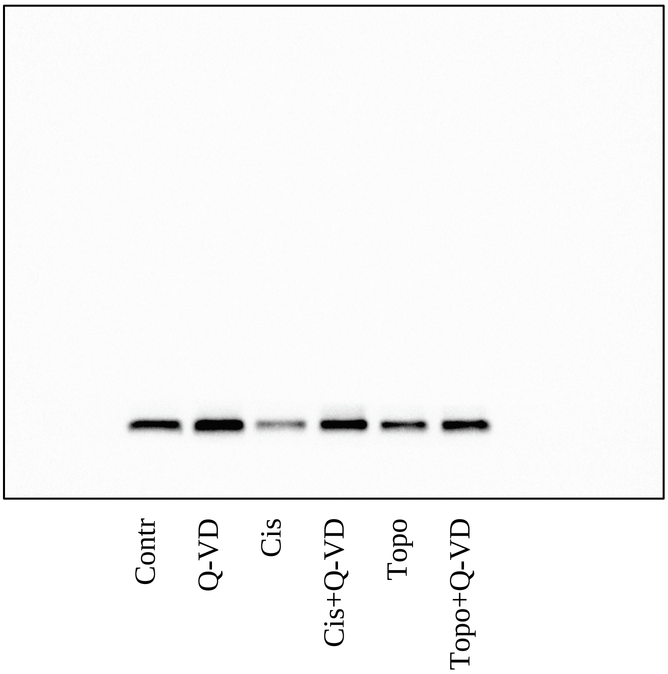

MAP1LC3B

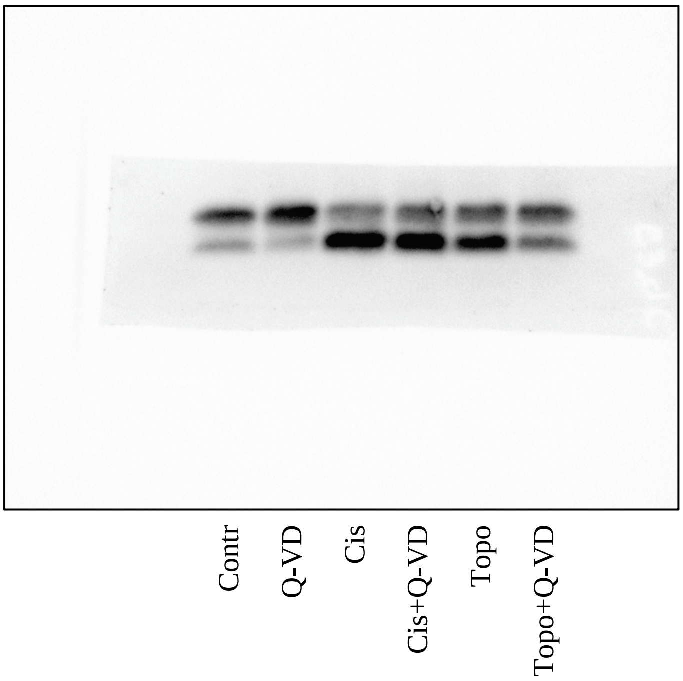

CASP3

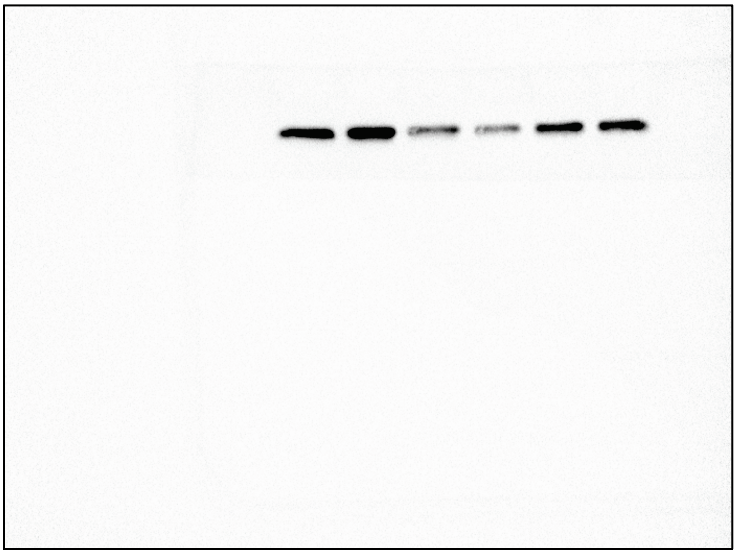

Contr
Q-VD
Cis
Cis+Q-VD
Topo
Topo+Q-VD

Cleaved CASP3

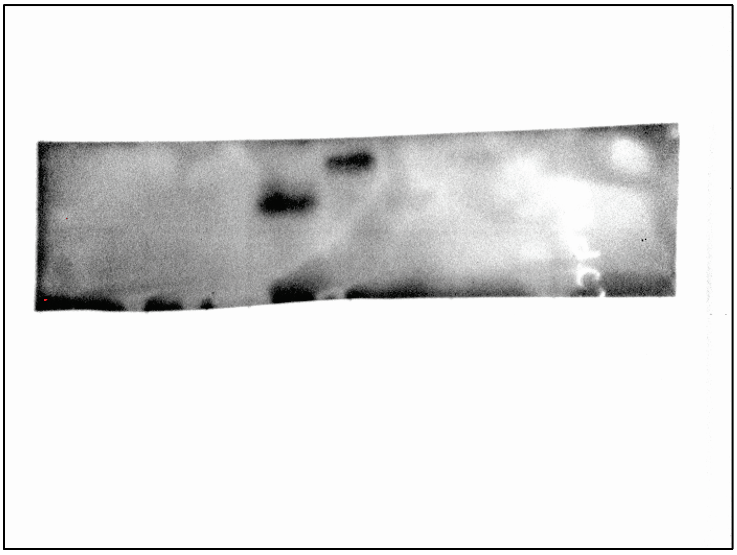

Contr
Q-VD
Cis
Cis+Q-VD
Topo
Topo+Q-VD

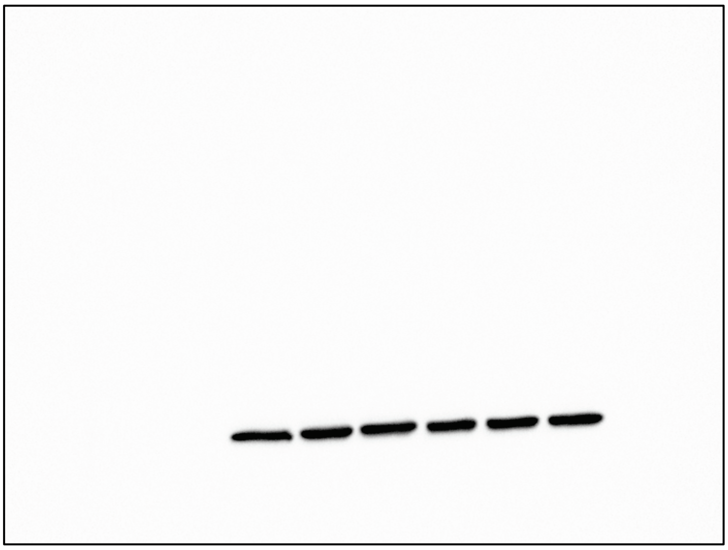
GAPDH
Contr
Q-VD
Cis
Cis+Q-VD
Topo
Topo+Q-VD

Figure 3d – SKOV3:

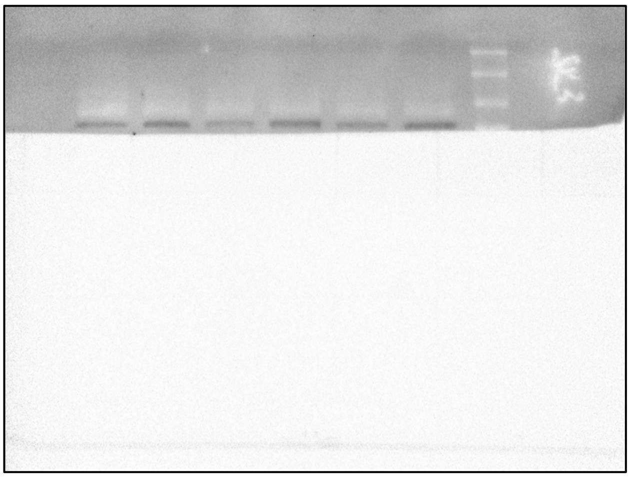

p-RIPK1 (S166)

Contr Q-VD Cis Cis+Q-VD Topo Topo+Q-VD

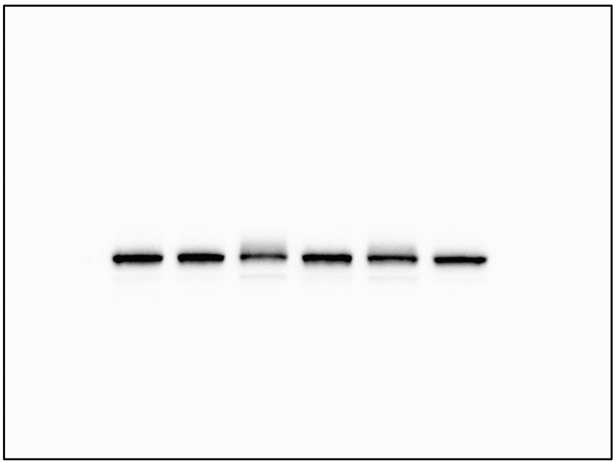

PARP1

Contr Q-VD Cis Cis+Q-VD Topo Topo+Q-VD

SQSTM1/p62

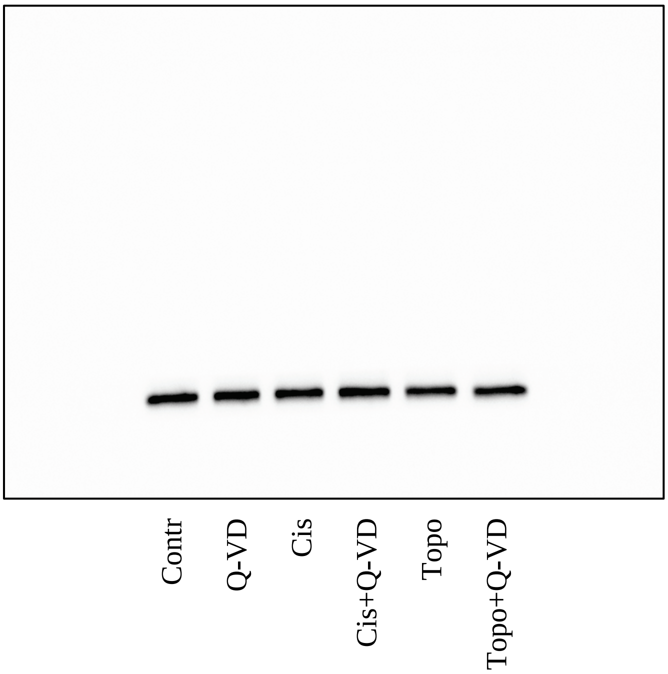

MAP1LC3B

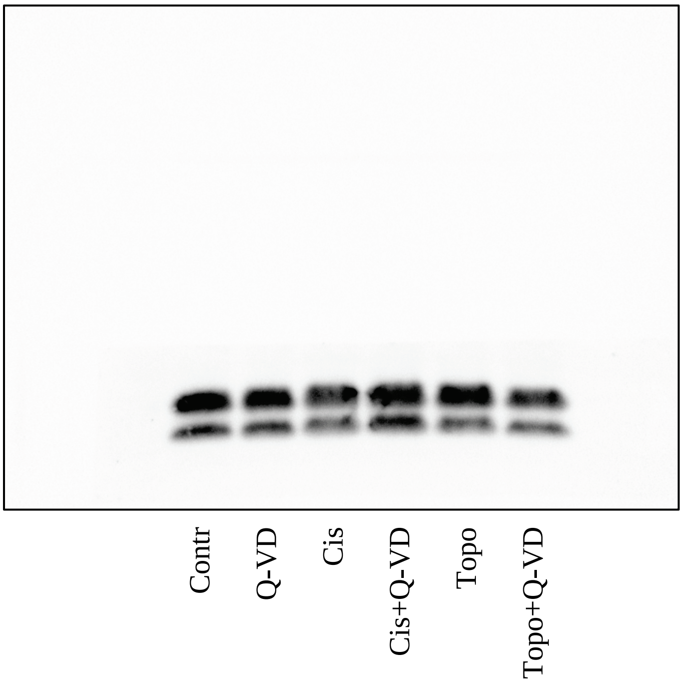

CASP3

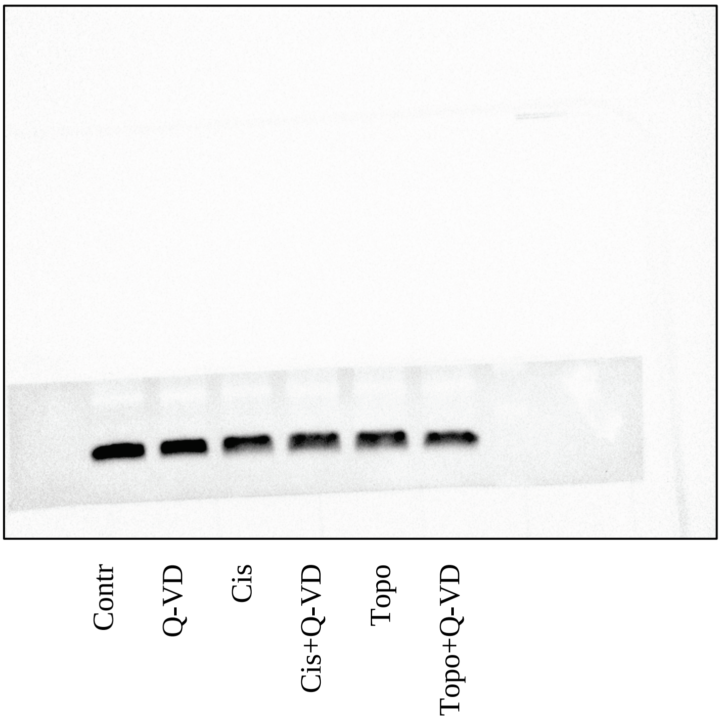

Cleaved CASP3

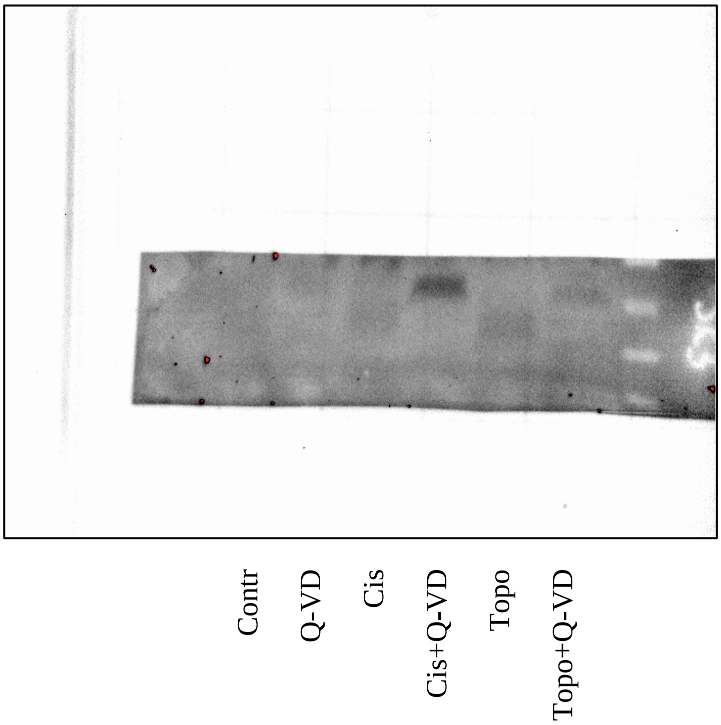

GAPDH

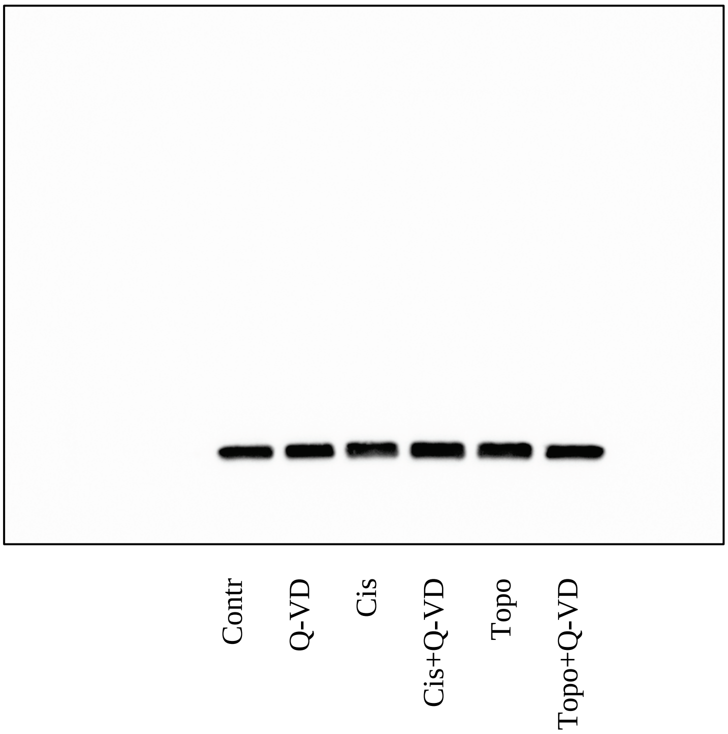

Supplementary Figure 4d:

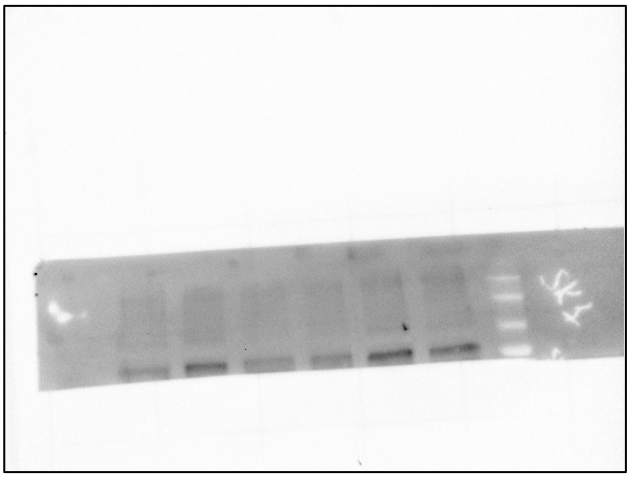

p-RIPK1 (S166)

Contr
Q-VD
Cis
Cis+Q-VD
Topo
Topo+Q-VD

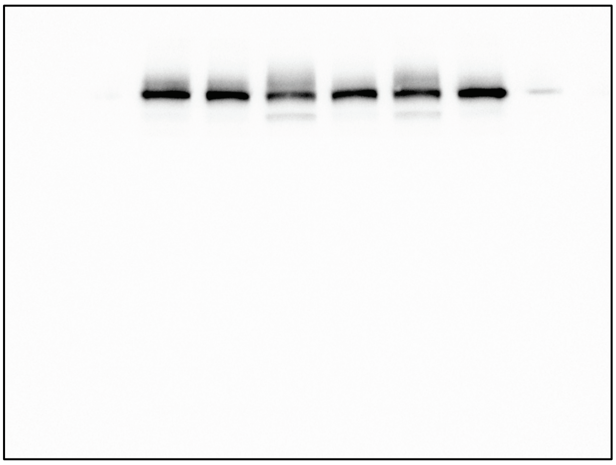

PARP1

Contr
Q-VD
Cis
Cis+Q-VD
Topo
Topo+Q-VD

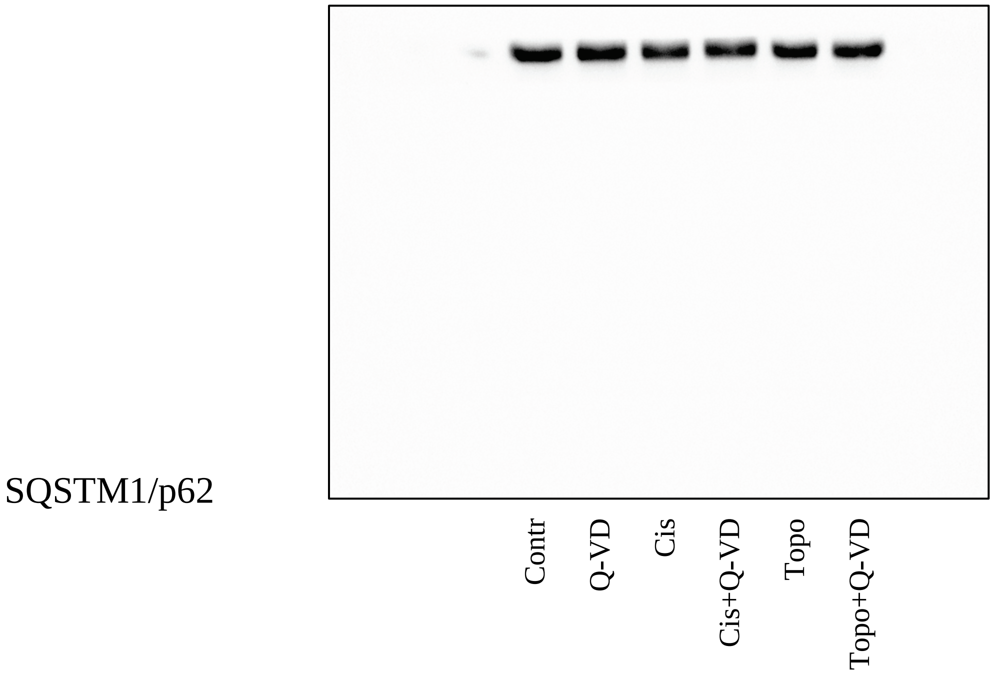
SQSTM1/p62
Contr
Q-VD
Cis
Cis+Q-VD
Topo
Topo+Q-VD

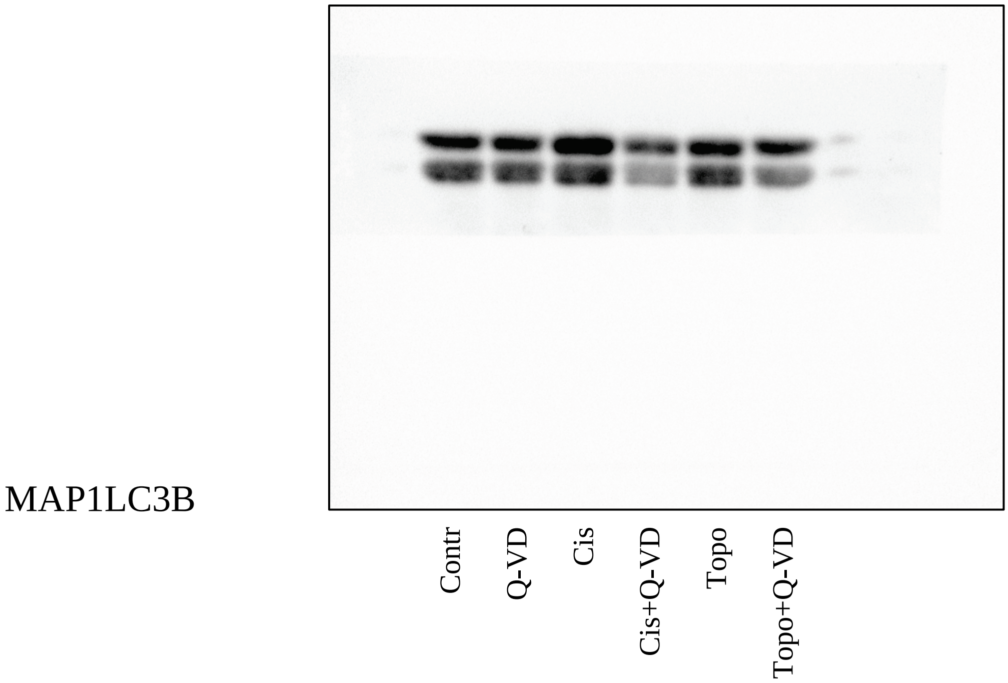
MAP1LC3B
Contr
Q-VD
Cis
Cis+Q-VD
Topo
Topo+Q-VD

CASP3

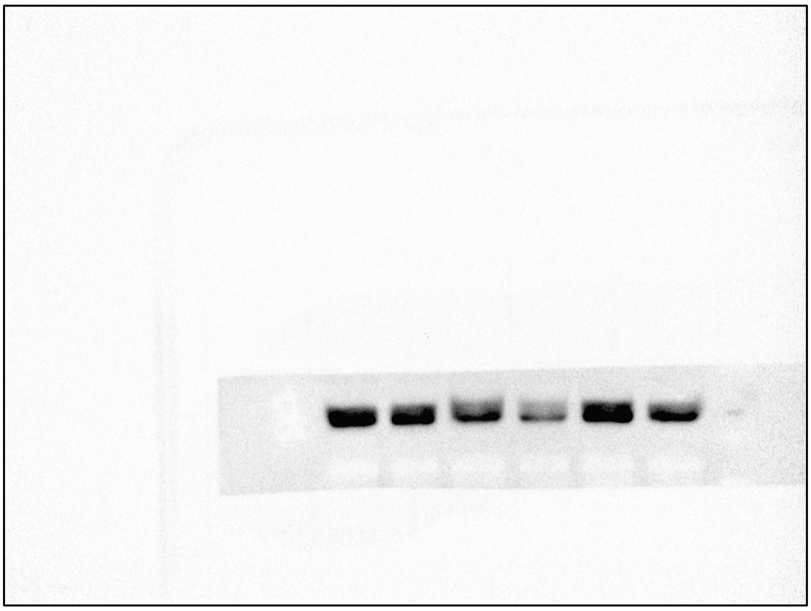

Cleaved CASP3

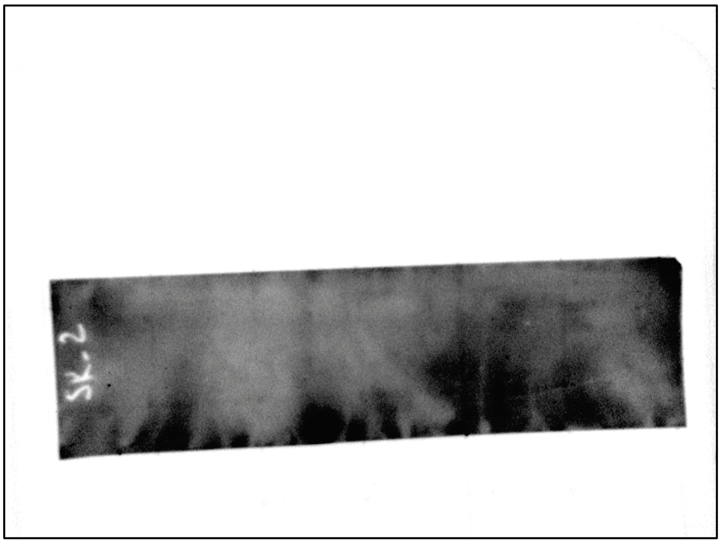

GAPDH

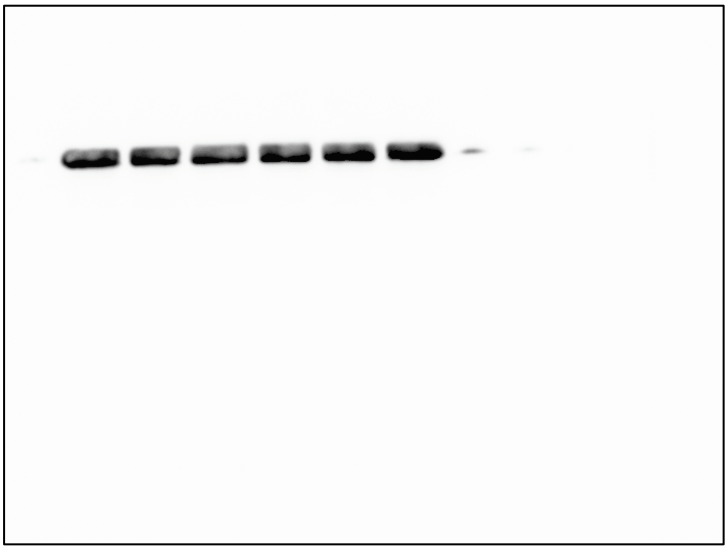

Contr

Q-VD

Cis

Cis+Q-VD

Topo

Topo+Q-VD
